# A 13,000-year history of vegetation and fire in a rare inland pine barrens: The Albany Pine Bush (Albany County, New York, USA)

**DOI:** 10.1371/journal.pone.0314101

**Published:** 2024-12-18

**Authors:** Megan Tremblay, J. Curt Stager, Jeannine-Marie St-Jacques, Skylar Murphy, Matthew Peros, Brian S. Carl

**Affiliations:** 1 Department of Geography, Planning and Environment, Concordia University, Montréal, Québec, Canada; 2 Department of Natural Sciences, Paul Smith’s College, Paul Smiths, New York, United States of America; 3 Faculty of Social Sciences, Bishop’s University, Sherbrooke, Québec, Canada; 4 Department of Earth and Environmental Sciences, State University of New York at Potsdam, Potsdam, New York, United States of America; Centre National de la Recherche Scientifique, FRANCE

## Abstract

Two radiocarbon-dated pollen and charcoal records from cores collected at Stump Pond and a wetland in suburban Albany County, New York, provide new insights into the environmental history of a unique inland pine barrens that is currently surrounded and threatened by urban development: the Albany Pine Bush (APB). The Stump Pond core shows that the pond formed roughly 13,000 years ago with the recession of glacial Lake Albany. From ca. 13,000 to 11,000 years ago spruce (*Picea*) and other boreal forest taxa were more common in the region than they are today, but both cores show that pine-oak (*Pinus-Quercus*) assemblages similar to those of today’s APB have been predominant components of the local forests for the last ca. 11,000 years. Abundant charcoal in both cores demonstrates that fire activity was a frequent occurrence in the APB throughout its history, particularly for the last ca. 6400 years. Water tables rose in response to increasingly humid hydroclimates, leading to the establishment of the wetland site ca. 6400 years ago and a greater abundance of ferns and mosses there during the last millennium. More recently, expanding urbanization and its associated impacts demonstrate that human activity has become the primary driver of change in the APB ecosystem.

## Introduction

**Fig 1:**
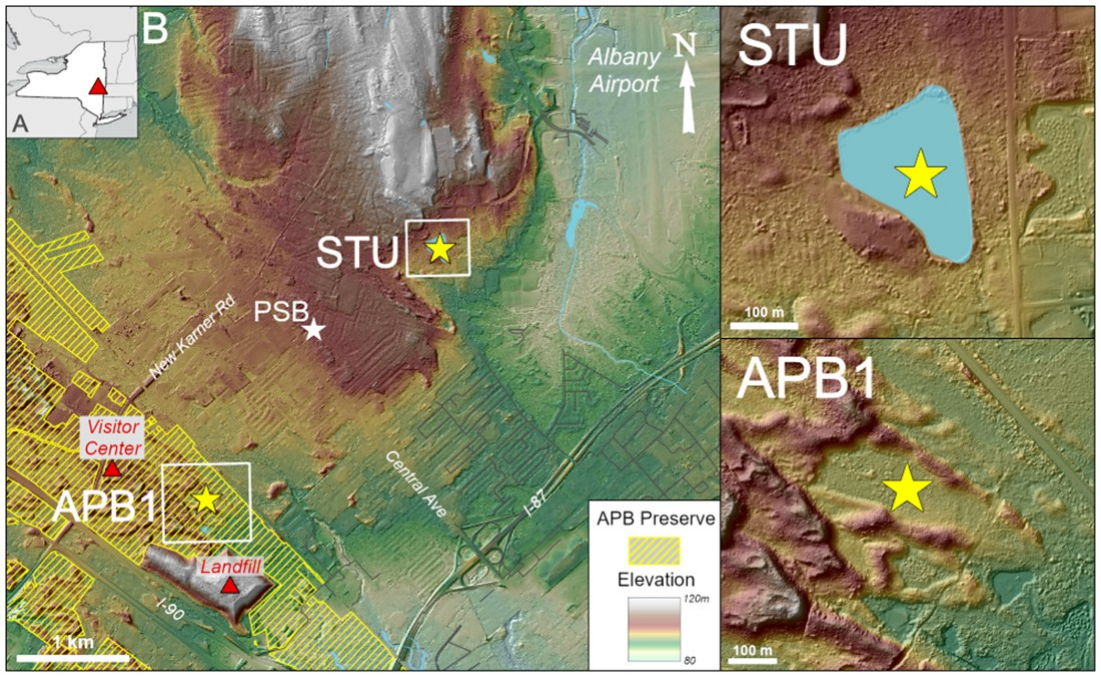
Site maps. A: Location of the APB (red triangle). B. Locations of coring sites (stars) and selected labeled landmarks on the western outskirts of Albany, New York. STU: Stump Pond coring site. APB1: Wetland coring site showing paleo-dune topography. PSB: Poplar Street Bog coring site [4]. Yellow cross-hatching represents property of the Albany Pine Bush Preserve. (base imagery from [6], accessed 5 April, 2024).

The Albany Pine Bush Preserve is a 1300 ha mosaic of mixed pine-deciduous woodland and heathlands embedded within the suburban outskirts of Albany, New York (Fig 1). It is both a popular recreational area and a refuge for rare and endangered insects including Karner blue butterflies (*Plebejus samuelis*), frosted elfins (*Callophrys irus*) and inland barrens buckmoths (*Hemileuca maia*) [1]. Characterized by a patchwork of fire-tolerant pitch pine (*Pinus rigida*), scrub oak (*Quercus ilicifolia*), and open heathland rich in grasses, shrubs, and wildflowers, the APB is the largest inland pine barrens ecosystem in North America [1]. Conservation and management of the Preserve requires rigorous protocols of controlled burning, removal of invasive species, and protection from urban encroachment that must also balance such needs against those of surrounding residents, businesses, and roadways [2, 3]. The success of these procedures requires a firm understanding of the ecology of the APB both past and present, but long-term perspectives on the ecosystem’s age, vegetational dynamics, fire history, and sensitivity to climatic change have thus far been difficult to obtain.

The physical setting of the APB has hindered reconstruction of its history prior to the colonial period. Lakes and peatlands which are the primary sources of long paleoecological records in the Northeast have been largely destroyed in the APB by land development, leaving scientists and managers to draw most of their historical inferences from similar ecosystems elsewhere. A peat core from one local wetland, Poplar Street Bog (Fig 1), yielded general insights into the APB’s vegetation history [4], but it lacked a radiometrically dated chronology and did not include detailed charcoal profiles. Unfortunately, the Poplar Street Bog site has since been destroyed by development, thereby preventing follow-up investigations at that site. In the absence of more definitive information, speculations about the age of the APB’s pyrogenic vegetation community have ranged from centuries to millennia, and hypotheses about its origins have included causes ranging from recent human activity to various combinations of soil, climate, and lightning-caused fires [1, 4, 5].

In this study, we present radiocarbon-dated pollen and charcoal records from two sites within or adjacent to the current boundaries of the APB Preserve that represent most of the last 13,000 years. A sediment core from Stump Pond, situated between the Preserve and Albany International Airport (Fig 1), provides insights into the post-glacial origin of the vegetational community and long-term changes in fire frequency. We supplement this post-glacial history with a peat core record from a wetland within the Preserve that represents the last ca. 6400 years. With these records of environmental history spanning the last 13,000 years we address the following basic questions: (1) How old is the APB’s oak-pine vegetation community? (2) How prevalent has fire been in the APB since its origin? (3) What role has climatic change played in the development of the APB ecosystem?

## Study site

### Physical setting

The underlying bedrock of the APB region is composed mainly of Paleozoic sandstones and shales [1, 7]. As is typical of pine barrens in coastal regions, the soils of the APB are mainly derived from sandy parent materials but, rather than being of marine origin, the APB sands were deposited ca. 15,000–14,000 years ago by the Mohawk River as a delta on the western rim of glacial Lake Albany, which formerly submerged what is now the Albany-Schenectady area [8]. The sandy delta deposits underlie an area of ca. 100 km^2^ between Albany and Schenectady at an elevation of roughly 100 m above mean sea level, representing the former boundaries of what was once a more extensive pine barrens ecosystem [1]. When crustal rebound and ice retreat caused Lake Albany to recede from the APB landscape ca. 13,000 years ago, the exposed deltaic sands created parabolic dune fields that were eventually stabilized by vegetation, forming irregular topography atop the otherwise relatively flat terrain (Fig 1) [1, 7–9].

The Stump Pond coring site is situated on a private residential property 3 km northeast of the APB wetland in the town of Colonie, New York (42°44’06”N, 73°49’42”W). It is small (2 ha), shallow (< 1.5 m), and triangular in shape (Fig 1). Although the pond is of natural origin, the current owner reported that a previous owner dredged an undetermined amount of surface sediment from the bottom of it to deepen it for aesthetic purposes (S. Gorbachev, personal communication 2021).

The ephemeral wetland from which the APB1 core was taken is an outcrop of the water table that is exposed in a depression between forested paleo-dune ridges (Fig 1). Its surface water level fluctuates considerably in response to seasonal and interannual variability in hydroclimatic conditions, and the APB1 coring site was damp, without standing surface water, when we sampled it in July, 2020. The wetland lies within the main body of the Albany Pine Bush Preserve (42°42’57”N, 73°51’17”W) ca. 800 m southeast of the visitor center, is ca. 200 m long, and covers an area of ca. 1.5 ha. The core was taken from the open, grass-covered mid-section of the wetland (Fig 1).

### Biotic setting

Typical woodland vegetation within the APB consists mostly of pitch pine, scrub oak, and dwarf chestnut oak (*Q*. *prinoides*) [1]. Scrub oaks and pitch pines are shade-intolerant early successional trees that are resistant to fire damage. Pitch pines have thick bark that protects them from surface fires and their serotinous cones release regenerative seeds after fire disturbances. Scrub oaks have large root systems that survive frequent, low-grade fires and allow the tree to take advantage of newly opened landscapes through rapid sprouting [10]. In portions of the APB where fire has been suppressed for long periods, the arboreal community is more diverse and includes white pines (*P*. *strobus*) and various hardwoods including maple (*Acer*) and birch (*Betula*) species [1].

Unforested areas are primarily maintained by fire and contain an assortment of prairie grasses and shrubs such as blueberry (*Vaccinium angustifolium*), huckleberry (*Gaylussacia baccata*), bracken fern (*Pteridium* sp.), and New Jersey tea (*Ceanothus americanus*), as well as lupines (*Lupinus perennis*), which are the primary host plant for endangered Karner Blue butterflies [1]. Management of the APB includes controlled burning to maintain open areas suitable for lupines and the rare insects who depend upon them. Vertebrates of the APB include New York state-listed animals of concern such as spotted turtles (*Clemmys guttata*) and blue-spotted salamanders (*Ambystoma laterale*), as well as birds and mammals with large biogeographical ranges who nonetheless find their primary habitats in the Albany-Schenectady area within the APB Preserve [1, 11].

### Cultural setting

Indigenous peoples have inhabited the APB region since deglaciation, and stone implements they left there represent multiple cultural phases that archaeologists have classified as Paleo-Indian, Archaic, and Woodland periods [12]. Indigenous residents and utilizers of the region during the last millennium included members of the Muhhekunneuw (Mohican) Nation and Haudenosaunee (Iroquois) Confederation [9, 13]. Beginning in the 16^th^ century, the Hudson Valley’s Euro-American colonizers removed substantial amounts of timber from the woodlands but the infertile sandy soils discouraged significant farming or other settlement until more recently [1, 14]. The APB and its immediate surroundings have experienced increasing residential and commercial development following the 1954 construction of the New York Thruway which, along with a network of smaller roads, cuts through the center of the barrens [15].

Today, the APB is an actively managed mosaic of public and private lands, now much reduced from a probable former extent of closer to 100 km^2^ [1]. Following the loss of roughly half of the APB to development and fire suppression between the 1950s and 1980s, a Preserve Commission was established in 1988 to protect the remaining barrens habitat [1, 15]. The commission’s management plans focus on preventing major successional change and the incursion of invasive plant species, including black locust (*Robinia pseudoacacia*) and tree of heaven (*Ailanthus altissima*), and employ methods such as pulling by hand, tree-girdling, mowing, and herbicide application [2, 3, 16, 17]. Since 1991, prescribed burning has also been used to mimic estimated natural fire cycles of ca. 10–40 years [2, 3, 10, 16]. The management guidelines allow for no more than 30% of the pitch pine-scrub oak area to be burned per year with a current average of 37 ha being either burned or mowed annually [15, 17]. Currently, about one third of the Preserve supports pitch pine-scrub oak forest, thicket, and open heathlands that are considered typical of the pre-colonial APB ecosystem [1, 17].

## Materials and methods

### Field methods

Peat and sediment cores were collected from the APB1 wetland and Stump Pond sites in July, 2020, with a hand-held Russian peat sampler capable of penetrating fibrous material. The Stump Pond core (STU, 0–48 cm) was collected by lowering the peat sampler from a canoe in ca. 1.5 m water depth. Thick rootstocks of water lily (*Nymphaea*) obstructed several trial attempts before the sampler penetrated soft organic pond sediment. That drive was halted by a dense mixture of sand and organic matter after collecting 48 cm of sediment. Permission to sample Stump Pond was granted by the owner, Sergey Gorbachev.

The APB1 core was obtained from the wetland surface in two sequential, adjacent drives, APB1-A (0–50 cm) and APB1-B (50–90 cm), before the sampler was halted by impenetrable material. An additional drive (APB2) at the same location confirmed that a dense layer prevented further penetration, and a soil auger sample identified the barrier as a dense, rusty hardpan that, if extensive, could contribute significantly to water retention in the wetland. Sampling of the wetland was conducted under a research permit provided by Neil Gifford, Conservation Director of the Albany Pine Bush Preserve Commission.

### Laboratory methods

Bulk sediment and peat samples of 1 cm^3^ volume were removed from selected depths in the STU, and APB1 and APB2 cores, respectively, oven dried, and delivered to the NOSAMS facility at Woods Hole, Massachusetts, for radiocarbon dating by accelerator mass spectrometry. Subsamples of fixed volume (see below) were also removed from the STU and APB1 cores for pollen and charcoal analysis. Age-depth models were created using the R package “rbacon” (version 3.0.0) [18] with the IntCal20 radiocarbon calibration curve [19], together with an estimated core-top date of 2020 for APB1-A with an error of 5 years. The accumulation rates were estimated at 1-cm resolution and were constrained by prior information on the accumulation rate (gamma distribution with a mean, “acc.mean,” and shape “acc.shape”), and its variability between neighbouring depths, or "memory" (beta distribution). For APB1, acc.mean = 50 years/cm, acc.shape = 1.5, mem.mean = 0.5, and mem.strength = 10. For STU, the settings were the same, except acc.mean = 100 years/cm.

Standard techniques were used to process the pollen samples [20, 21]. Core subsamples of 0.6 cm^3^ volume were immersed in both HCl and KOH, sieved, and subjected to acetolysis to eliminate excess organic material. One Lycopodium tablet was added to each of the 105 subsamples before processing, staining with safranin, and immersion in silicone oil. At least 300 pollen grains and spores were identified per sample at 1000X magnification. Regional pollen guides [22–24] were used as references. Pollen was identified to genus with the exception of fern spores belonging to different genera which were combined into the category “Ferns” (Class Polypodiopsida). Pollen grains of pine (*Pinus*) were identified as belonging to the haploxylon and diploxylon groups when possible, or otherwise categorized as “*Pinus* spp.” Pollen of pitch pine closely resembles that of red pine (*P*. *resinosa*), but because the latter taxon is rare to absent in the APB today we have categorized such pollen as pitch pine in this study. Only terrestrial pollen and spores were used to calculate the final pollen sum.

Pollen stratigraphic plots of relative abundance were made with the R package “rioja” and CONISS was used to divide them into zones whose significance was assessed by the broken stick test [25–27]. To further examine changes in the taxa through time, principal components analysis (PCA) was performed for each record using the R package “vegan” [28]. We down-weighted the abundance of the most numerous taxa by using a *log(x+1)* transformation applied to all taxa to prevent the dominant taxa from overwhelming the analyses. Similar results were obtained using a square-root transformation and are not reported further. The gradient length was checked using the length of the first axis of DCA to ensure that PCAs were appropriate, and the significance of the first two axes was checked with a broken stick model.

Charcoal analyses were also conducted using standard methods [29, 30]. Contiguous core subsamples of 1 cm^3^ volume (1 cm thickness) were deflocculated in an 8% sodium hypochlorite and 5% sodium hexametaphosphate solution for at least 24 hours, then sieved through a 150-micron mesh filter. Charcoal particles were identified by manually sorting the remaining sediments using a fine-tipped paintbrush under a dissecting microscope. A digital image of the charcoal particles in each sample was taken through the microscope, and each image was analyzed with WinSEEDLE^®^ software (Regent Instruments, Inc.) to determine the surface areas and numbers of particles caught on the filters.

The non-parametric Spearman’s correlation test was used to assess the relationship between charcoal surface area and charcoal counts. Because charcoal particles can fragment, the analysis was subsequently restricted to charcoal surface areas. The analysis for each core was done with the Matlab CharAnalysis package [31, 32] which interpolated the raw data to the median sample resolution bin size, because sample resolution was quite different between the two cores. The background charcoal records (BCHARs) of non-local and secondary charcoal were calculated using moving mode smoothers and moving windows of 900 years for both cores to maximize the signal-to-noise index (SNI) of the charcoal peaks to a level above 3.0 [31, 32]. The BCHAR series represent distant fires, long-term shifts in fire regimes and secondary taphonomic processes not related to local fire occurrences (e.g. sediment mixing, intra-lake macrocharcoal transport, slope wash). For each core, BCHAR was subtracted from the interpolated surface areas to form the residual of high-frequency peaks CHAR. Peaks in charcoal interpreted as local fire events were identified as CHAR values exceeding the 95^th^ percentile of the CHAR noise distribution modelled using Gaussian mixture models with locally set thresholds.

## Results

### Chronology

Between the two cores, the age-depth models covered a time frame of approximately 13,000 calibrated years. The STU core record spanned ca. 13,000–7200 calibrated years before present (cal yr B.P.), and the APB1 core record spanned ca. 6400 cal yr B.P. to the present, leaving a temporal gap of roughly 8 centuries between them (Table 1). One subsample from 5 cm depth in core section APB1-B yielded a radiocarbon age that appeared to be anomalously young and was therefore omitted from the age-depth model. Although care was taken to maintain stratigraphic integrity between the adjacent, sequential APB core drives, it is possible that the young age of the 5 cm sample in APB1-B represented a slight stratigraphic overlap between the two APB1 cores. We therefore consider the age model for the uppermost 10–15 cm of APB1-B to be tentative. We also lacked radiometric dates for the top of core APB1-A, so our estimate of “zero” age for the youngest subsample is only approximate.

**Table 1:** Radiocarbon ages of samples and modern coretop date from the STU (Stump Pond) and APB1 (wetland) cores. Maximum, minimum, and median calibrated 2-σ age ranges shown here were calculated with CALIB version 8.2 [19]. For the APB1-B core, the sample depths are listed as within-core depths with total combined depths in parentheses. “B.P.” refers to years before A.D. 1950. The age obtained for the 5 cm sample in APB1-B (italics) was considered to be an outlier and was not included in the APB1 age-depth model. Not applicable is denoted by *n*.*a*. for the APB1 coretop.

Depth (cm)	^14^C age (yr B.P.)	Maximum-minimum calibrated age (B.P.)	Median age (B.P.)	NOSAMS Sample #
**APB1-A**				
**0**	n.a.	n.a.	-70 ± 5	n.a.
**10**	970 ± 20	794–926	850	161904
**25**	1780 ± 20	1612–1718	1655	161905
**40**	2820 ± 20	2859–2993	2920	161906
**APB1-B**				
***5 (55)***	*2510 ± 25*	*2493–2725*	2585	*161907*
**20 (70)**	4240 ± 25	4654–4857	4830	161908
**35 (85)**	5440 ± 30	6197–6296	6235	161909
**STU**				
**2**	6510 ± 25	7328–7479	7420	162017
**5**	7860 ± 30	8549–8771	8635	162018
**10**	8710 ± 35	9545–9885	9645	162019
**15**	9340 ± 45	10,409–10,694	10,550	157231
**30**	9770 ± 35	11,163–11,249	11,210	162020
**45**	10,650 ± 50	12,501–12,734	12,680	157232

**Fig 2:**
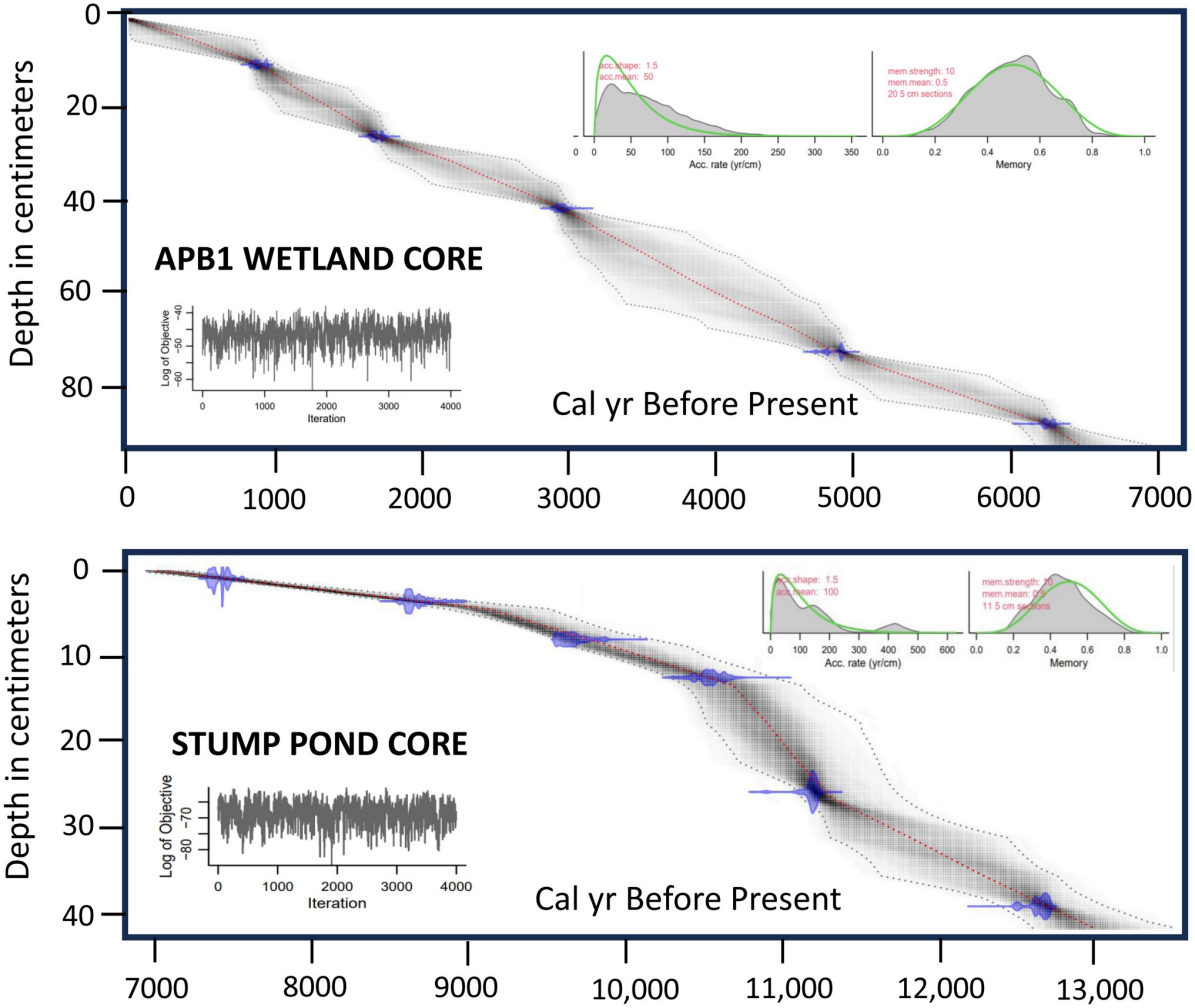
Age-depth models for the STU and APB1 cores. The time scales spanning ca. 13,000 cal yr B.P. to the present were obtained using the “rbacon” package in R [18]. Probability ranges of the radiocarbon dates are shown in purple. The central red dashed lines shows the ‘best” models based on the weighted mean age. The outer dashed lines denote 95% confidence levels.

The age-depth profile of the STU pond core displayed more internal variability than that of APB1, with more rapid sediment accumulation rates indicated for the lowermost 27 cm of the core (Fig 2 and S1 Fig). The median sample resolution was 99 years with a range of 34 to 418 years. The inferred mean sediment accumulation rate in the lower section of STU was 0.014 cm/yr, but it decreased to 0.004 cm/yr in the uppermost 14 cm of the record. Sedimentation rates were much more regular in the APB1 core (Fig 2 and S2 Fig). The median sample resolution for the APB1 core was 70 years with a range of 50 to 99 years, and the mean sedimentation rate was 0.014 cm/yr.

### Pollen records

**Fig 3:**
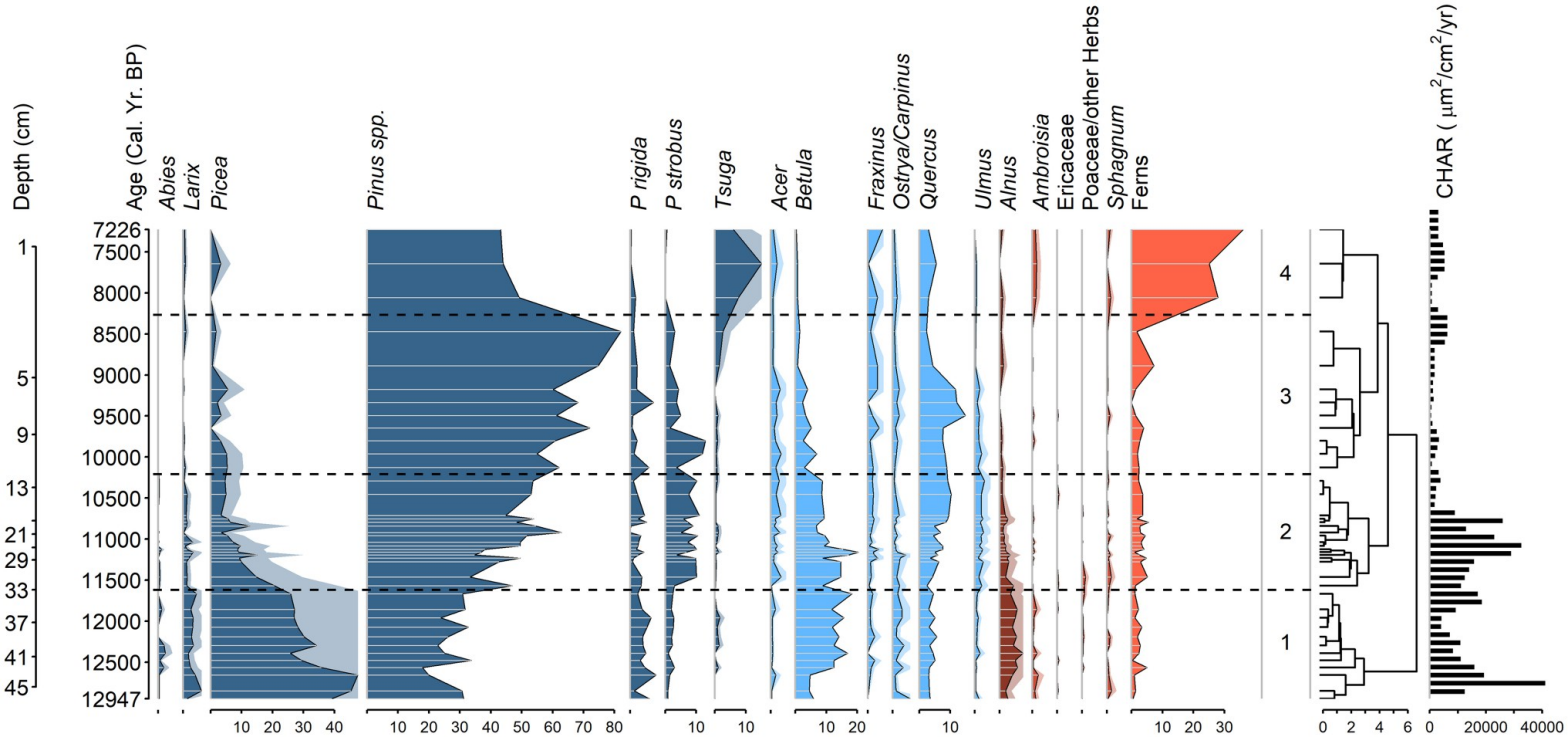
Pollen and charcoal profiles from core STU (ca. 13,000–7200 cal yr B.P.). Values are given as percentages of total terrestrial pollen grains and fern spores. Depths and calibrated ages of sediment subsamples are indicated along the left margin. Zonation was performed using CONISS with significance tested by a broken stick model. Interpolated charcoal influx units are in μm^2^/cm^2^yr.

**Fig 4:**
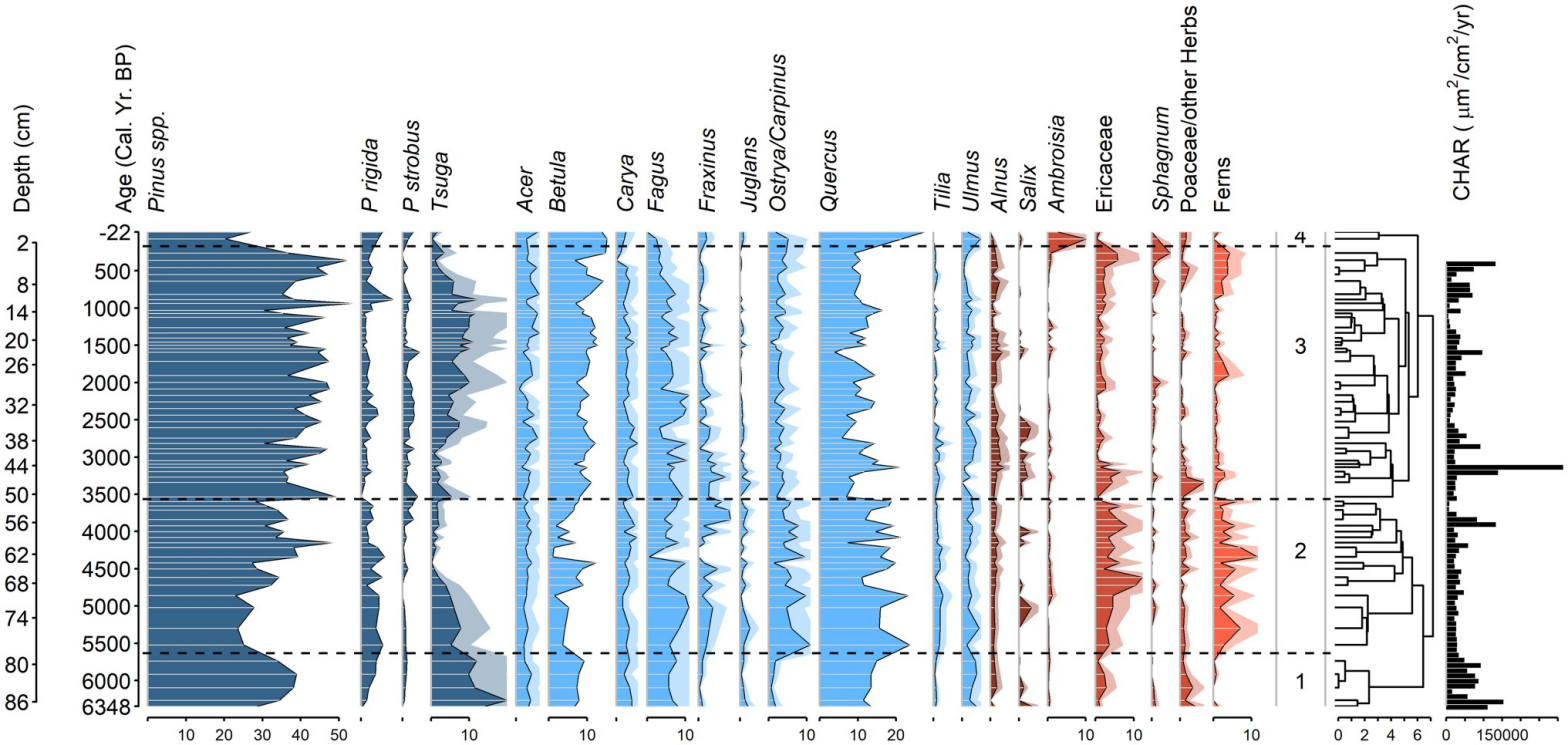
Pollen and charcoal profiles from wetland core APB1 (ca. 6400 cal yr B.P. to present). Values are given as percentages of total terrestrial pollen grains and fern spores. Depths and calibrated ages of peat subsamples are indicated along the left margin. Interpolated charcoal influx units are in μm^2^/cm^2^yr.

Among the 105 samples analyzed, 31 pollen and spore taxa were identified, but we focus here only on the most visually obvious and ecologically informative features of the two records. Pine pollen in general, most of which could not be reliably identified to species due to variable preservation, was the most abundant type found throughout both cores with a mean abundance of 40% (Figs 3 and 4). The two taxa that most clearly define the pitch pine-scrub oak barrens today, pitch pine and oak, were present throughout both cores as well, with mean percentages of 3% and 10%, respectively. Little aquatic pollen was present. Both cores had four significant pollen assemblage zones, as assessed by the broken stick model.

In pollen zone 1 of the STU pond core (47–33 cm; ca. 13,000–11,700 cal yr B.P.), spruce pollen was abundant (26–48%), and larch (*Larix*), alder (*Alnus*), birch, pine spp., and pitch pine were lesser dominants with a low but sustained presence of fir (*Abies*) (Fig 3). In pollen zone 2 (33–12 cm; ca. 11,700–10,300 cal yr B.P.), spruce declined fairly steadily to 5% at the top of the zone. Indeterminate pine spp. and eastern white pine (*P*. *strobus*) rose throughout this zone, and pine spp. became the dominant taxon. Birch and alder declined while oak, elm (*Ulmus*), and maple increased. In pollen zone 3 (12–3 cm; ca. 10,300–8400 cal yr B.P.), pine spp. reached its maximum (82% at ca. 8500 cal yr B.P.), as did eastern white pine (13% at ca. 9800 cal yr B.P.) and oak (15% at ca. 9500 cal yr B.P.). Eastern white pine, birch, and elm declined, while eastern hemlock (*Tsuga canadensis*) increased. In pollen zone 4 (3–0 cm; ca. 8400–7200 cal yr B.P.), both eastern hemlock pollen and fern spores (Polypodiopsida: mainly *Osmunda*, *Dryopteris* and *Cystopteris* spp.) were exceptionally numerous, and although pine spp. was less abundant than in zone 3, it remained the dominant taxon.

In the APB1 wetland core, pine spp. was the dominant pollen taxon (mean of 38%) and pitch pine was present in low percentages (mean of 3%) in all samples (Fig 4). Oak was the secondary dominant and was common in all samples (ca. 10–20%). The heath family (Ericaceae), grasses (Poaceae), and other herbs were consistently present throughout the core in minor amounts, suggesting the presence of open forest canopy. Pollen zone 1 (88–79 cm; ca. 6400–5700 cal yr B.P.) was characterized by dominant pine spp., oak, and eastern hemlock, with birch and beech as minor taxa. Pollen zone 2 (79–51 cm; ca. 5700–3600 cal yr B.P.) included elevated amounts of the heath family, ferns, and hornbeam/hop-hornbeam (*Ostrya/Carpinus*), as well as a steep decline of eastern hemlock, which reached its lowest value at 63 cm (ca. 4400 cal yr B.P.). Examination of pollen influx data (not shown) showed that the apparent increases in pitch pine, heath family, ferns, grasses, and herbs during the prolonged hemlock decline were primarily due to the sharp decrease in hemlock percentages in zone 2. Pine spp. and oak were consistent dominants, with subdominants of pitch pine, beech, birch and hickory (*Carya*). Pollen zone 3 (51–3 cm; ca. 3600–200 cal yr B.P.) included the highest sustained percentages of pine spp. and maple in the APB1 core, a slow partial recovery of eastern hemlock, and sub-dominant oak and birch. In pollen zone 4 (3–0 cm; roughly 1800 AD to present; the Euro-American zone), ragweed (*Ambrosia*) pollen reached its highest percentages of 3–10% in the uppermost two samples. Pollen influx data (not shown) indicated that ragweed pollen influx did not increase in zone 4; rather, the influx of other taxa decreased. Oak also reached its maximum abundance there (27%).

**Fig 5:**
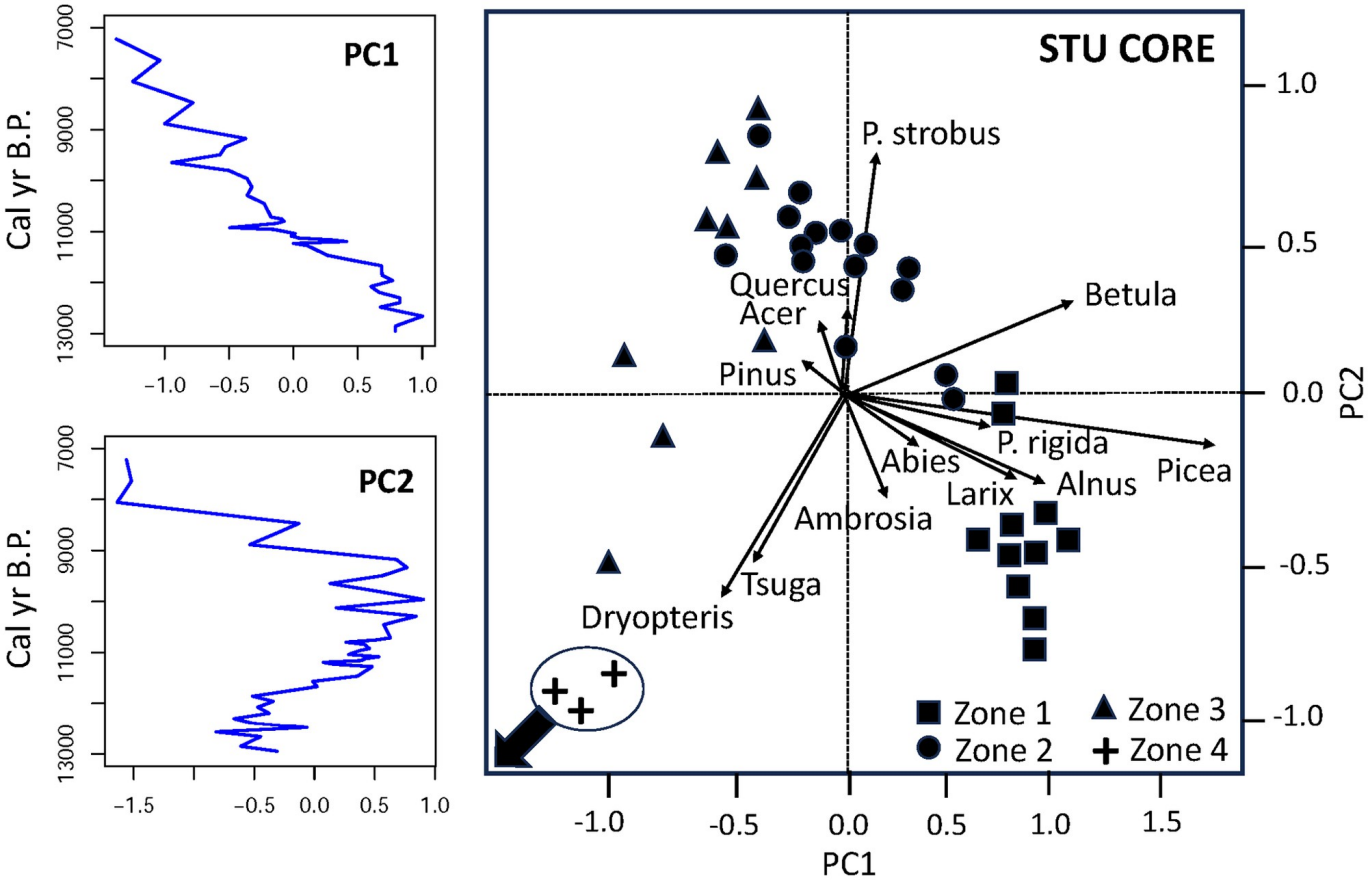
Principal components analysis results for core STU. Left: Plots of first and second PCA axes versus time. Right: The biplot for the first two principal components shows taxa (arrows) and samples assigned to four pollen zones with CONISS (shapes). Arrow: Zone 4 samples (circled) were positioned off the scale to the lower left (PC1 values -1.1 to -1.4; PC2 values ca. -1.6).

In the STU core, PCA axis 1 explained 46% of the variability in the pollen record (Fig 5). It was defined by spruce, birch, alder, and larch (positive scores) and by eastern hemlock and ferns (negative scores). PCA axis 1 scores decreased in the samples that were deposited during the post-glacial climate warming period, so this axis might reflect temperature variations. PCA axis 2 explained 20% of the variability in the STU core and was defined by eastern white pine, maple, elm, and oak (positive scores) and ragweed, ferns and eastern hemlock (negative scores). As eastern hemlock is a late successional species, this axis might reflect succession.

**Fig 6:**
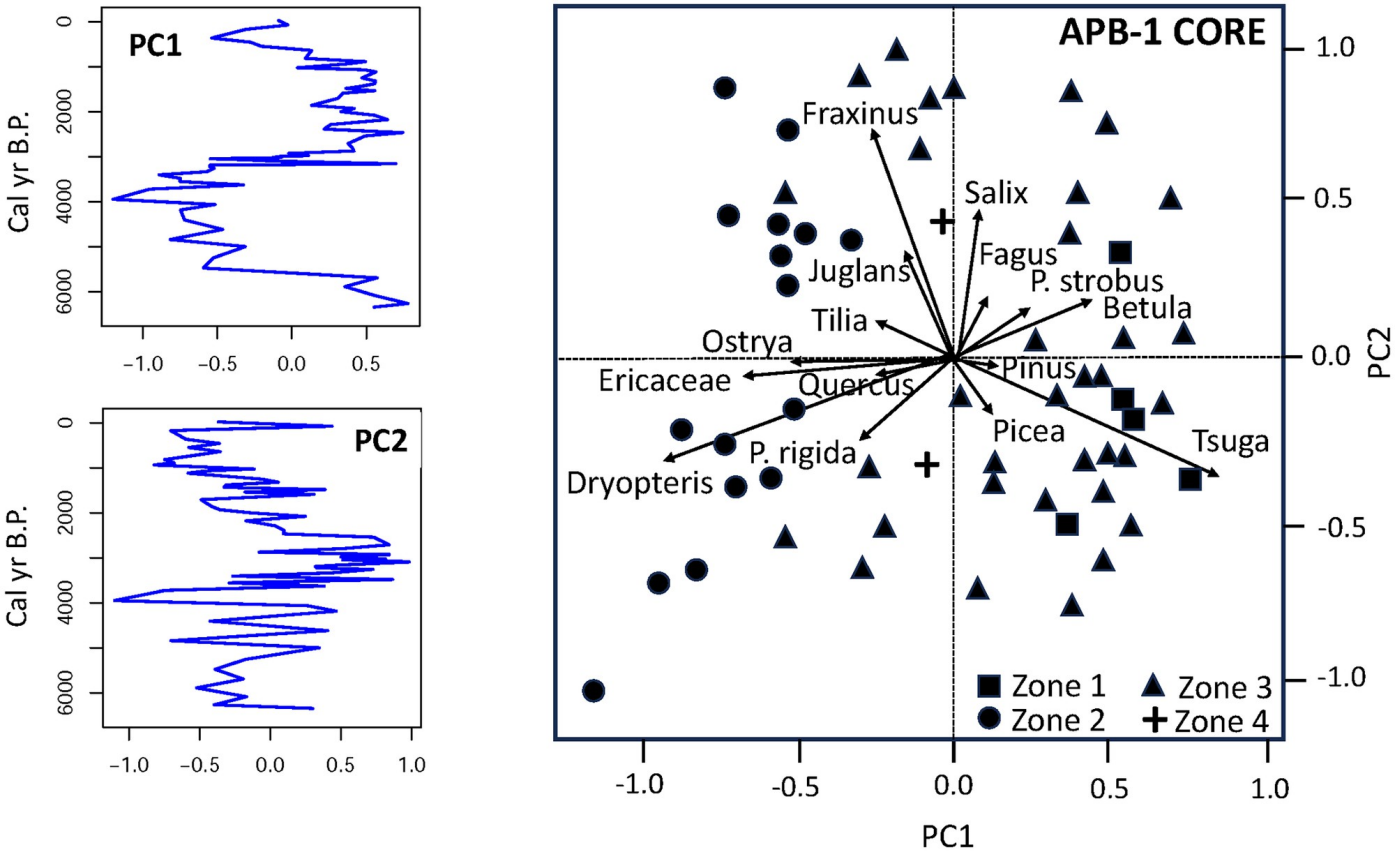
Principal components analysis results for core APB1. Left: Plots of first and second PCA axes versus time. Right: The biplot for the first two principal components shows taxa (arrows) and samples assigned to four pollen zones with CONISS (shapes).

In the APB1 core, PCA axis 1 explained 25% of the variability in the pollen record (Fig 6), and it separated the subsamples according to their percentages of eastern hemlock and birch (positive scores) versus pitch pine, heath family, hornbeam/hop-hornbeam, and ferns (negative scores). The first PCA axis suggests a fire-frequency gradient, with fire intolerant eastern hemlock together with birch forming one end of the gradient and more fire tolerant pitch pine/heath/ferns forming the other end. PCA axis 2 explained 13% of the variability in the core and was defined by ash (*Fraxinus*), willow (*Salix*), and walnut (*Juglans*) (positive scores) and pitch pine (negative scores). PCA axis 2 appears to segregate moisture-adapted lowland taxa (ash, willow, walnut) from pitch pine, which might suggest a moisture gradient associated with landscape position (for example, lowland vs. upland or interdune swale vs. dune ridge).

### Charcoal records

**Fig 7:**
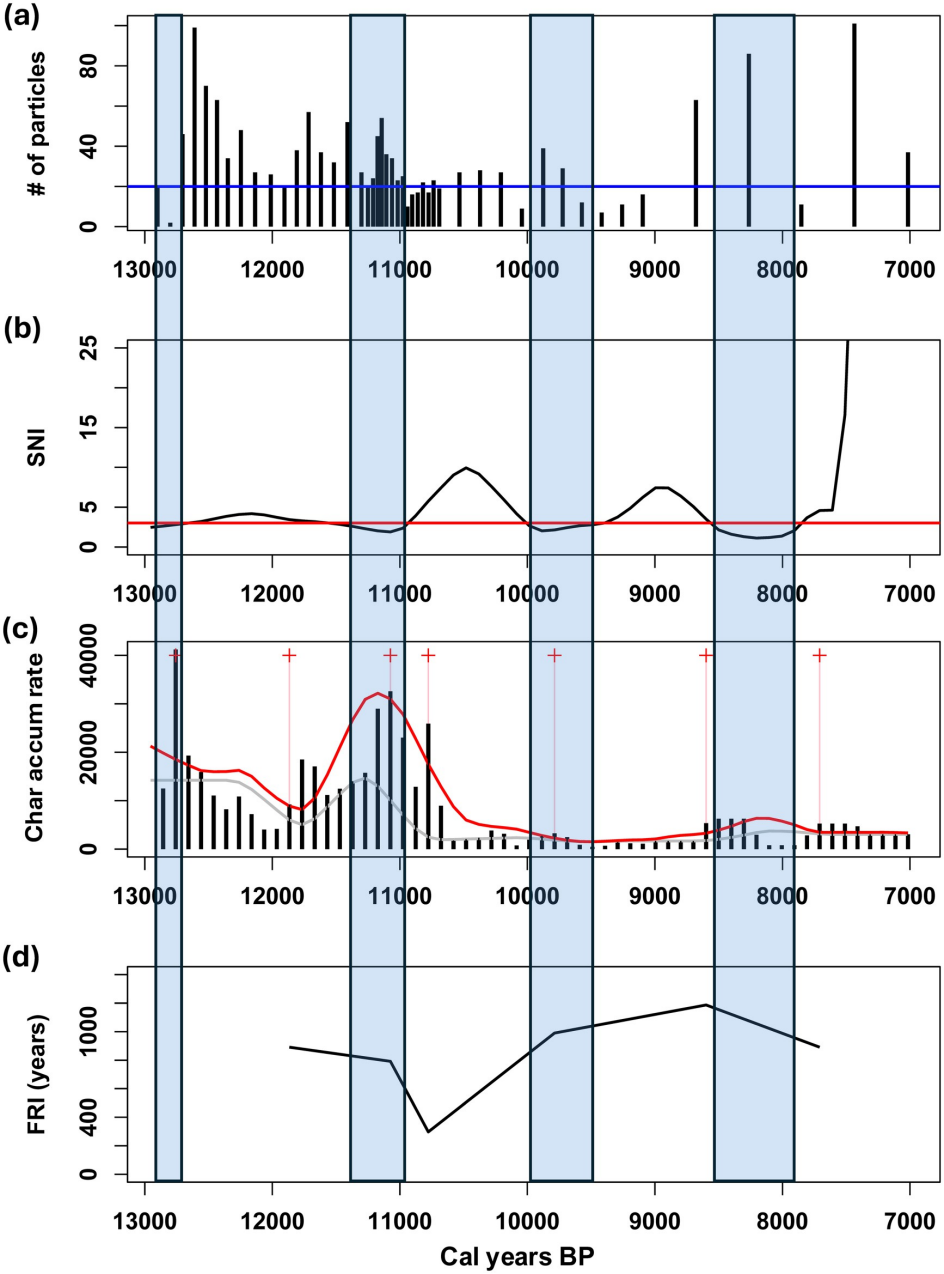
STU fire record for 13,000–7,000 cal yr B.P. (a) Plot of the number of raw charcoal particles per sample. (b) Plot of the smoothed signal to noise index (SNI), together with a red line at 3, the SNI cut-off. (c) Plot of the interpolated charcoal accumulation rate (μm^2^·cm^-2^·yr^-1^) record (black bars). Local fire peaks are marked by red crosses and pink vertical lines. The grey line shows background charcoal (BCHAR) and the red line marks the 95% threshold of the Gaussian noise models. (d) Plot of fire return intervals (FRIs). The blue transparent rectangles denote where the SNI < 3, where results should be treated with caution.

Spearman’s rank correlation test indicated significant relationships between the surface areas and charcoal particle numbers for the STU core (ρ = 0.60, p = 6.9 x 10^−6^). Because of the highly variable sedimentation rate in STU, the samples were unevenly distributed temporally (Fig 7a). Twelve of the 47 samples had counts < 20. The SNI was ≥ 3 for 12,700–11,600, 10,900–10,100, 9,400–8,600, and 7,800–7,000 cal yr B.P., representing 72% of the record (Fig 7b). CharAnalysis identified 7 peaks in STU, 4 of which occurred in regions with SNI ≥ 3 (Fig 7c). The local fire peaks were slightly more abundant in pollen zones 1 and 2 representing boreal forest (13,000–10,300 cal yr B.P.) than in the later portion of the record, and the interpolated charcoal accumulation rates were higher in pollen zones 1 and 2, as well (1.4 x 10^4^ μm^2^·cm^-2^·yr^-1^ versus 2.8 x 10^3^ μm^2^·cm^-2^·yr^-1^, respectively). The mean FRI over the entire record was 842 years, and in the sections where the SNI ≥ 3, the mean FRI was 1040 years (Fig 7d). This is equivalent to 1.2 and 1.0 fires per millennium, respectively.

**Fig 8:**
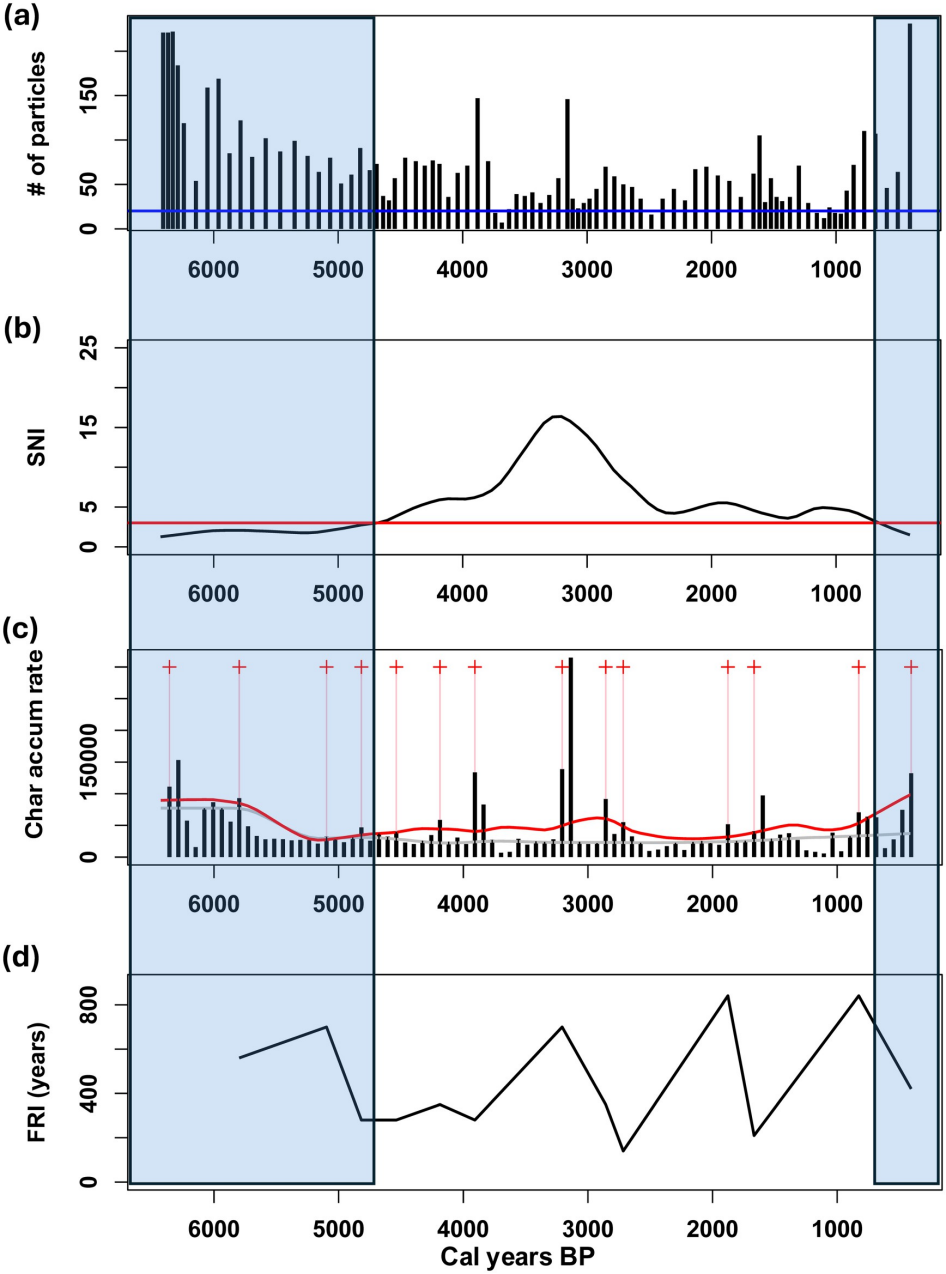
APB1 fire record for 6,400–500 cal yr B.P. (a) Plot of the number of raw charcoal particles per sample. (b) Plot of the smoothed signal to noise index (SNI), together with a red line at 3, the SNI cut-off. (c) Plot of the interpolated charcoal accumulation rate (μm^2^·cm^-2^·yr^-1^) record (black bars). Local fire peaks are marked by red crosses and pink vertical lines. The grey line shows background charcoal (BCHAR) and the red line marks the 95% threshold of the Gaussian noise models. (d) Plot of fire return intervals (FRIs). The blue transparent rectangles denote where the SNI < 3, where results should be treated with caution.

Spearman’s correlation test also indicated a significant relationship between surface areas and charcoal particle numbers for the APB1 core (ρ = 0.86, p < 2.2 x 10^−16^), for which 85 charcoal samples were counted. Charcoal contents in APB1 varied from 7 to 231 pieces per subsample (Fig 8a) except for the top 5 cm of the core (ca. 400 cal yr B.P. to present) which contained hundreds of times more charcoal than the other subsamples (S3 Fig). We therefore omitted the uppermost 5 cm of the core from subsequent calculations because of their overwhelming dominance of the records. Overall, more charcoal was found in the APB1 core than in the STU core (means of 3.1 x 10^6^ μm^2^/cm^3^ and 1.0 x 10^6^ μm^2^/cm^3^, respectively) (Figs 7a and 8a). The SNI was ≥ 3 between 6450 and 680 cal yr B.P. (Fig 8b). CharAnalysis identified 14 local fire peaks in APB1 which were more or less regularly distributed in the record (Fig 8c). The mean FRI between 6450 and 680 cal yr B.P. was 443 years (Fig 8d), equivalent to 2.3 fires per millennium.

## Discussion

The pollen and charcoal records of the STU and APB1 cores allow us to address the three main questions under investigation in this study. First, the association of oak and pine in APB forests has persisted continuously within the region since the end of the deglacial period ca. 11,000 years ago (Figs 3 and 4). Even during the preceding interval 13,000–11,000 cal yr B.P. when spruce, larch, alder, and fir were more abundant than today, pines (including pitch pine) were common and oaks were present despite their low pollen abundance. The presence of grass and heath family pollen throughout the APB1 record (Fig 4) indicates that open heathlands similar to those found in today’s APB were also continuously present on the landscape during the last ca. 6400 years.

Second, fires have occurred in the region throughout the last 13,000 years. At STU Pond, ca. 13,000–7200 years ago, the mean fire return interval was ca. 840 years (Fig 7). Because of its varying sedimentation rates and sampling intervals (S1 Fig), we have less confidence in the accuracy of this local fire record. The mean FRI during the last 6400 years at the APB1 site, with its steadier sedimentation rate, was 458 years (Fig 8 and S2 Fig). However, the limited temporal resolution of both charcoal records means that fire activity could also have been more frequent than these estimates.

**Fig 9:**
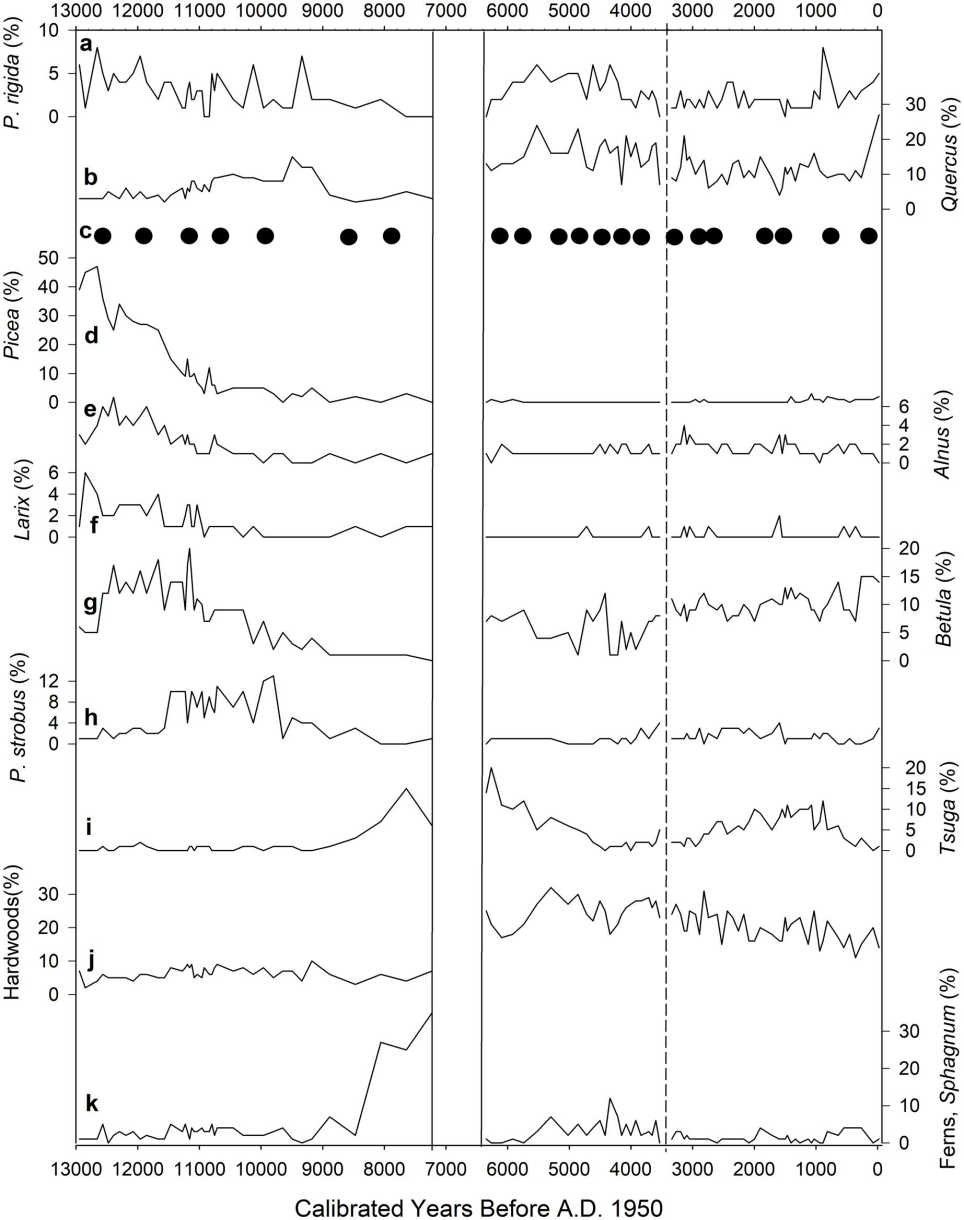
Vegetation assemblages and fire activity in the Albany Pine Bush region during the last ca. 13,000 years. Solid vertical lines represent the temporal gap between the records from the STU and APB1 coring sites. Dotted vertical line represents the period in which the APB1-A and APB1-B records meet. Solid circles represent significant peaks of fire activity (see Figs 7 and 8).

Third, the abundant deposition of spruce, larch, alder, and fir pollen at Stump Pond ca. 13,000–11,000 years ago (Figs 3, 5 and 9) suggests that climates in the APB were cooler than today during the late deglacial period. Pollen zone I of STU coincided with the Younger Dryas cooling event (ca. 13,000–11,700 cal yr B.P.) [33] which was also associated with high abundances of spruce elsewhere in New York and New England [9, 34–37]. In much of the Canadian Maritimes and Maine, tree abundances declined and vegetation reverted to tundra types [38, 39], but the effects of the cooling were generally less severe at inland locations in New England and New York [37, 40], including the APB. High sediment accumulation rates at Stump Pond during that time frame (Fig 2) suggest that wet climatic conditions might have increased erosional inputs to the pond then. However, mobile dunes in the vicinity could also have contributed to high sediment inputs, and the subsequent stabilization of dunes and local soils by vegetation could be consistent with reduced sedimentation rates after ca. 10,600 cal yr B.P.

The rise of eastern hemlock and fern percentages ca. 8000–7000 cal yr B.P. in the STU record (Figs 3 and 9) might reflect increasingly moist hydroclimates. Hemlock’s significance as a climate indicator in the APB1 record is complicated by the widespread hemlock decline of uncertain origin [41], which began in the APB1 pollen record roughly 5500 years ago and approached zero abundance ca. 4400 cal yr B.P. (Figs 4 and 9). However, the onset of wetland deposition at the APB1 site ca. 6400 years ago more clearly demonstrates that local water tables rose after the early Holocene, most likely due to increasingly wetter climates in the Northeast [42, 43]. Similarly, increased deposition of *Sphagnum* spores during the past several centuries (Fig 4) reflects a more recent rise in the water table. Today, artificial hydrological disturbances related to paved surfaces, groundwater extraction, and other direct anthropogenic factors act independently of climate and could put the APB wetlands and their biota at greater risk of desiccation and associated vulnerability to fires in future.

Several analytical factors limit the accuracy of our pollen records. Different plant taxa produce very different amounts of pollen and the various types of grains can travel vastly greater or lesser distances from the host plant, so the percentages of pollen types in a core need not clearly reflect species abundances in the adjacent vegetation [44–46]. For example, pine and birch pollen percentages tend to over-represent tree abundances while spruce and maple are under-represented. Furthermore, the seemingly anomalous radiocarbon age near the top of APB1-B might represent a slight stratigraphic overlap with the base of APB1-A, so we consider the chronology of the composite record within the ca. 4000–3000 cal yr B.P. time interval to be tentative. Anomalous radiocarbon ages in cores such as these are not unexpected and can result from a wide variety of causes including sediment disturbance by animals, root penetration, variable deposition rates within the wetland, or analytical error. On the other hand, the STU and APB1 sites are located relatively close to one another, neither site has major tributary streams that would affect pollen transport from distant source areas, and their soil types and elevations are similar so we feel justified in combining their records into a single, generalized composite. In light of the aforementioned limitations we focus only on general patterns, such as presence-absence and large overall changes in percentages, to reduce the risk of over-interpreting our results. We are therefore confident in concluding simply that pine-oak forests typical of the APB have been in place for approximately 11,000 years.

### Comparisons with other records

To our knowledge, the only other pollen record obtained from the APB was collected from the Poplar Street Bog (Fig 1B) [4]. This record lacks a radiometric age model that would facilitate comparisons to our own time series, but qualitative similarities between the pollen stratigraphies of the PSB, STU, and APB1 cores allowed us to infer a basic chronology for the former record (Figs 3 and 4). The APB1 core yielded ca. 6400 years of peat within 1 m, which suggests a possible age of roughly 13,000 years for the 2 meter-long PSB core. Spruce pollen is present, albeit in low percentages (< 5%), in the basal sediments of the PSB core as, it was in STU, which is consistent with a late deglacial age for the bottom of PSB. Finally, a notable decrease in eastern hemlock pollen occurs in samples above 90 cm depth in the PSB core and above a similar depth (80 cm) in APB1 (Fig 4). The mid-Holocene hemlock decline is thought to have occurred synchronously across the geographic range of this species and the onset of the rapid drop in hemlock pollen percentages in these cores can be used to represent a time interval of roughly 5500–5000 cal yr BP [41, 47, 48]. We therefore consider it likely that the PSB core represented a time period that was roughly equivalent to that of the STU and APB1 records combined.

Assuming that the 13,000-year time scale for the PSB core is correct, then the continually high abundances of pine and oak pollen in that record are consistent with our finding that forests typical of today’s pine barrens have been present in the APB since deglaciation, and certainly for the last ca. 6400 years. Charcoal was not quantified in the PSB core, but it was common in most subsamples [4], which is also consistent with our findings, and an increase of wetland pollen percentages in the upper half of the PSB core supports our conclusion that local water tables have risen in recent millennia.

Our pollen records from the APB are consistent with post-glacial pollen and paleoclimate records from other sectors of the northeastern United States, as well [7, 34, 41, 47–49]. Pollen and plant macrofossils from a peat deposit in the Mohawk River Gorge in Cohoes showed that spruce, pine, birch, and fir were common near the APB 12,700 to 11,000 years ago [9]. Pollen analysis of a core from Ballston Lake, in nearby Saratoga County, documented the presence of boreal forest rich in spruce, larch, and fir ca. 13,000–11,000 cal yr B.P., the subsequent development of mixed temperate forest with taxa such as hemlock, pine oak, and beech, and a decline of hemlock roughly 5000 years ago [34] that is also registered in a survey of sites from eastern North America as beginning ca. 5600 cal yr B.P. [41]. The basal age of the Stump Pond core is likewise consistent with the evidence from Ballston Lake, the peat deposit in Cohoes, and a 13,000-year-old mastodon skeleton that was recovered near the peat deposit [9] that the APB region had emerged from the waters of Lake Albany by ca. 13,000 cal yr B.P.

Most pine barrens in New England are generally thought to be of Holocene duration. A pollen record from a coastal pine barrens on Cape Cod yielded results similar to those found in the inland APB, with spruce- and alder-dominated assemblages being replaced by pine and oak-dominated assemblages ca. 11,000 years ago [50]. Long-term persistence of the pine-oak forest community since then, as indicated at Cape Cod and in the APB, is also documented in pollen profiles from the extensive coastal pine barrens of New Jersey [51]. A possible exception to that pattern comes from a pollen record of the last 6000 years from a small pine barrens near Rome, New York, which suggested that this habitat might be relatively young because pitch pine was uncommon there relative to hardwoods and “undifferentiated pines” prior to the 16^th^ century [5, 52].

Proxy-based reconstructions of Holocene climates in the Northeast typically indicate a climatic inflection point around 8000–7000 cal yr B.P. during which rising post-glacial temperatures peaked and then gradually decreased, while annual precipitation totals and/or effective moisture continued to increase into recent times [53–56]. The regional Holocene wetting trend probably contributed to the initiation of peat deposition at the APB1 coring site ca. 6400 years ago and the increase of fern and *Sphagnum* abundances there during recent centuries (Figs 3 and 4).

### Fire history of the APB

The charcoal records of Stump Pond and the APB1 wetland show that wildfires occurred frequently in the APB throughout its post-glacial history, particularly during the last 6400 years (Figs 7 and 8). However, the mean fire return values we obtained probably underestimate the true fire frequencies due to the relatively low sampling resolution and variable sedimentation rates indicated in our records (for example, 38–117 yr/cm in APB1). In time-series analysis of sediments, the highest fire frequency that can theoretically be detected in a charcoal record is the Nyquist frequency, for which estimates typically range from 1/(2 x sampling interval) [57] to 1/({4–5} x sampling interval) [D.J. Thomson, Queen’s University, *pers*. *comm*.]. As a result, our sampling intervals limited our ability to detect more frequent fire events in the environmental history of the APB. Nonetheless, the STU and APB1 records show that fire was a more or less constant feature of the landscape, particularly during the past 6400 years, and that such fires were likely to have contributed to the long-term persistence of pitch pine-oak-heathland communities in the APB.

The reproducibility of charcoal records can be limited by several factors, including sampling resolution, sample volume, and the uneven dispersal of charcoal fragments in sediments [58, 59]. We used a surface area-based charcoal record to avoid uncertainties related to fragment breakage and problems with the reproducibility of charcoal particle counts, but because of our limited sampling resolution and volumes, the Nyquist frequency, and varying sedimentation rates in the cores, we were only able capture a low-frequency overview of the APB fire record. Nonetheless, our charcoal records are able to demonstrate that fires were frequent enough to approach the Nyquist frequency, and the available charcoal and pollen evidence is strongly in favor of the long-term presence of a pyrogenic pine barrens at the APB. The analytical methods we used did not permit detailed insights into the extent, intensities, or causes of most of the fires registered in the cores, but further investigations could include using charcoal morphology to better understand changing fuel sources [60].

People were present in the APB region throughout the Holocene [12], but because many aspects of their fire-related behaviors and population sizes during that long time frame are unknown, the relative contributions of climate and human activity to fire history in the APB remain unclear. Historical and proxy records show that fires set by Indigenous people for hunting and/or horticultural purposes during the last millennium influenced forest communities in the general vicinity of the APB [5, 10, 61] and elsewhere in the Northeast [52, 61–67], although some investigations have suggested otherwise [68–70]. However, mobile hunting-gathering lifestyles prevailed in the APB region throughout most of its human history, and intensive use of fire in land clearance for horticulture was not widespread until the last millennium or so when Indigenous maize-based farming became common. The exceptionally high abundance of charcoal in the uppermost centimeters of the APB1 core was accompanied by increased percentages of *Ambrosia*, a sign of extensive land clearance [71] which, despite the lack of radiometric age control for the top of the core, does suggest that human activity intensified fire dynamics in the APB dramatically during the last few centuries.

Overall, the frequency of major charcoal peaks in our records remained more or less constant in the APB1 core despite increasingly moist hydroclimates that progressively raised water tables and allowed the APB1 wetland to form around 6400 years ago (Figs 4 and 9) [42, 43]. The STU and APB1 pollen and charcoal records establish a useful historical context for conditions within the APB in more recent centuries, during which heavy settlement and resource extraction have influenced vegetation communities and fire regimes more than human activities or climatic fluctuations of the deeper past.

## Conclusions

The APB ecosystem originated roughly 13,000 years ago, when spruce and other taxa typical of cool boreal forests were more common than today. A pine-oak vegetational assemblage including fire-tolerant pitch pine has dominated the forest community for the last ca. 11,000 years. Charcoal records from Stump Pond and the APB1 wetland suggest that major peaks of fire activity occurred there more or less continuously, particularly during the last 6400 years. Rising water tables due to a long-term wetting trend led to the formation of the APB1 wetland ca. 6400 years ago and to greater abundance of ferns and *Sphagnum* in the wetland in recent centuries. However, modern environmental changes in the APB including artificially variable fire frequency, vegetational shifts, and fluctuating water tables are now primarily anthropogenic, making humans an increasingly dominant force in the ecosystem today.

## Supporting information

S1 Fig Sampling integration times for the STU (Stump Pond) core. Plot of the sample integration times (yr/sample) from 13,000–7200 years BP. The red horizontal line indicates the median sampling integration time of 99 years for the entire core.
(TIF)

S2 Fig Sampling integration times for the APB1 (wetland) core. Plot of the sample integration times (yr/sample) from 6400 years BP to present. The red horizontal line indicates the median sampling integration time of 70 years for the entire core.
(TIF)

S3 Fig Example of charcoal content from omitted sample in the APB1 (wetland) core. The photographs were taken under the microscope from the sample at 2–3 cm depth, showing the large amounts of charcoal present.
(TIF)

S1 File (DOCX)
