# Supplementary figures and images for: A 13,000-year history of vegetation and fire in a rare inland pine barrens: The Albany Pine Bush (Albany County, New York, USA)
Source: PLoS One. 2024 Dec 18;19(12):e0314101. doi: 10.1371/journal.pone.0314101 (PMC11654978; doi:10.1371/journal.pone.0314101)

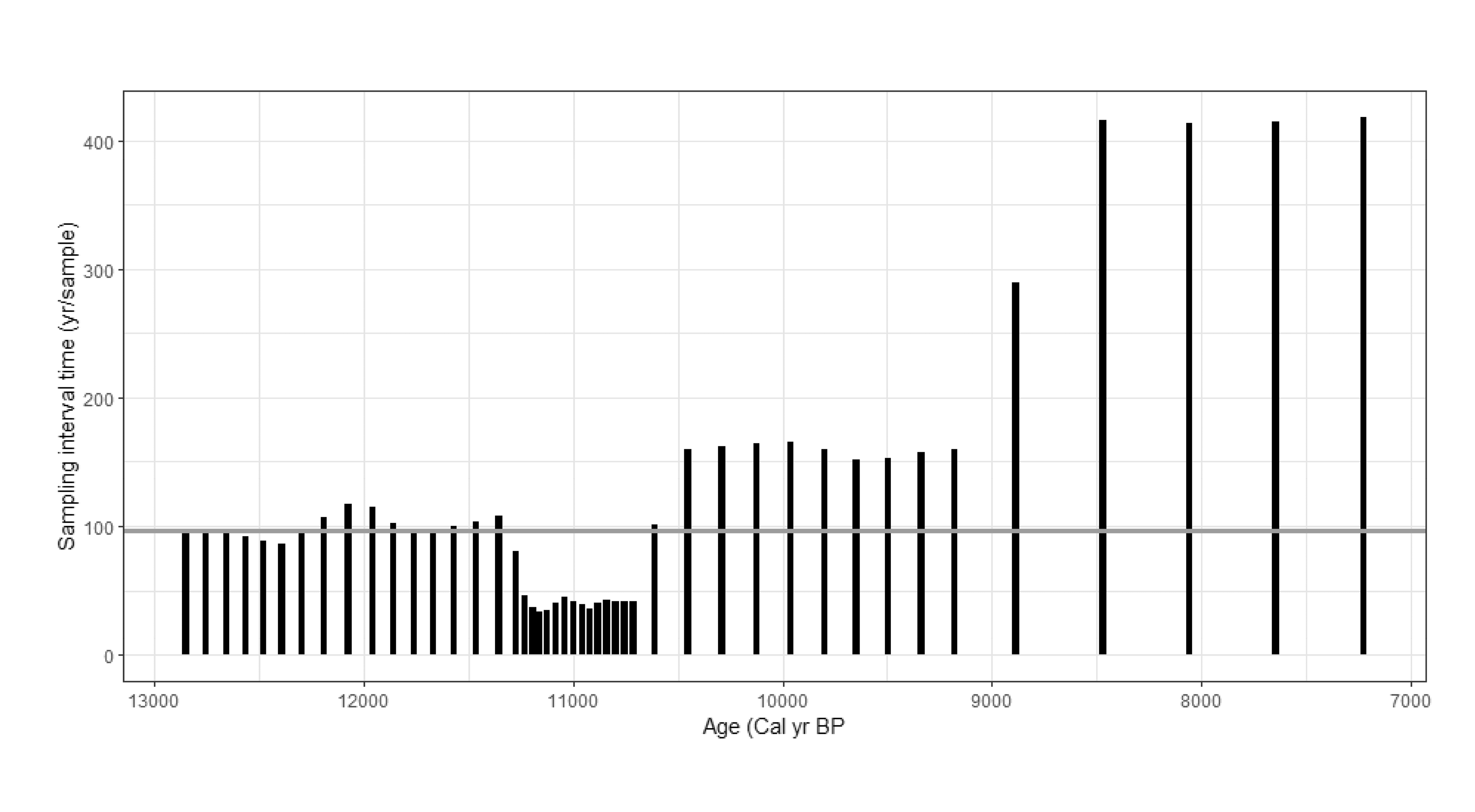

Supplement: S1 Fig — Plot of the sample integration times (yr/sample) from 13,000–7200 years BP. The red horizontal line indicates the median sampling integration time of 99 years for the entire core. (TIF) [file pone.0314101.s001.tif]

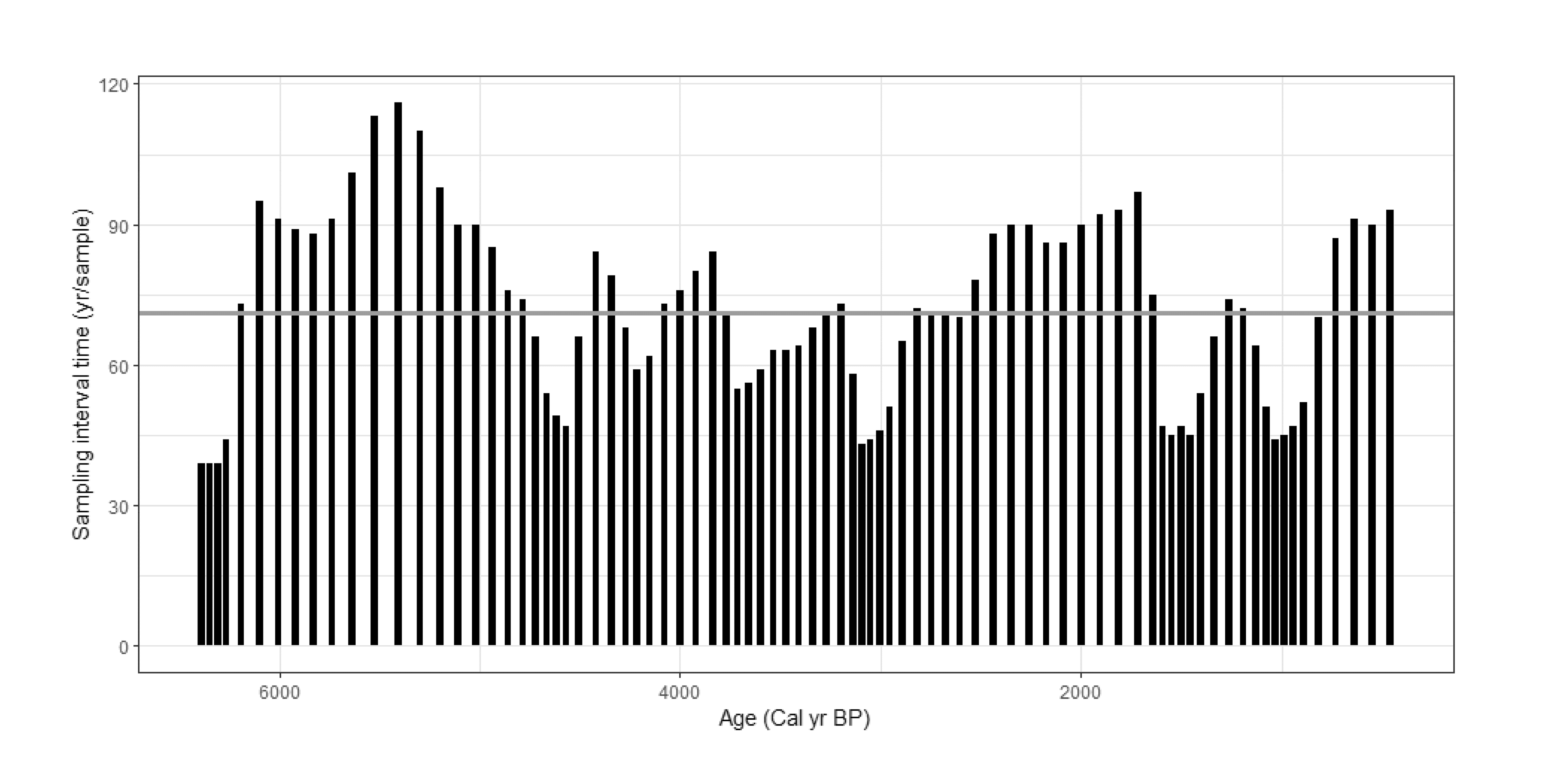

Supplement: S2 Fig — Plot of the sample integration times (yr/sample) from 6400 years BP to present. The red horizontal line indicates the median sampling integration time of 70 years for the entire core. (TIF) [file pone.0314101.s002.tif]

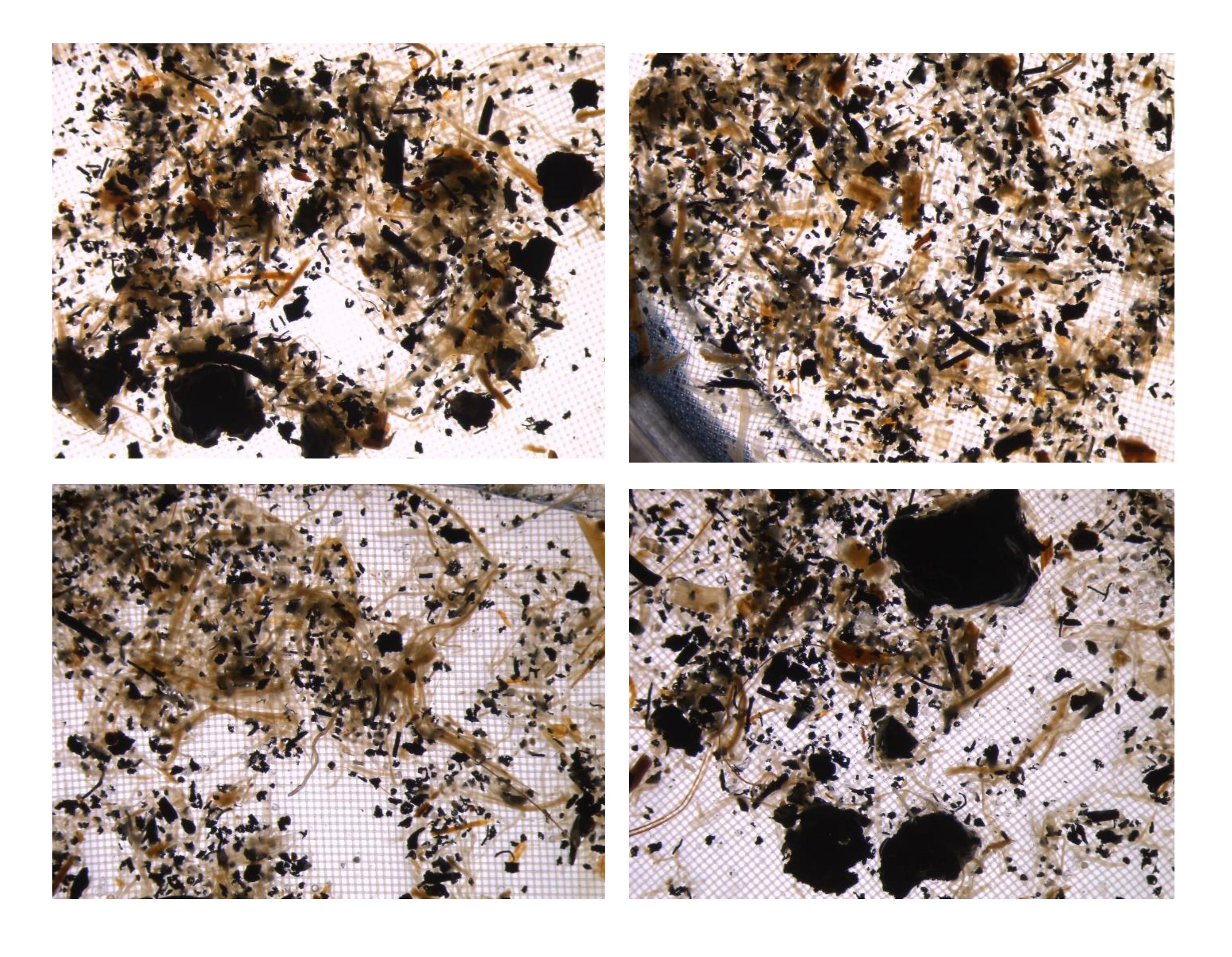

Supplement: S3 Fig — The photographs were taken under the microscope from the sample at 2–3 cm depth, showing the large amounts of charcoal present. (TIF) [file pone.0314101.s003.tif]
