# Supplementary material for: A 13,000-year history of vegetation and fire in a rare inland pine barrens: The Albany Pine Bush (Albany County, New York, USA)
Source: PLoS One. 2024 Dec 18;19(12):e0314101. doi: 10.1371/journal.pone.0314101 (PMC11654978; doi:10.1371/journal.pone.0314101)
Supplement: S1 File — (DOCX) [file pone.0314101.s004.docx]

**Supporting Information**

**
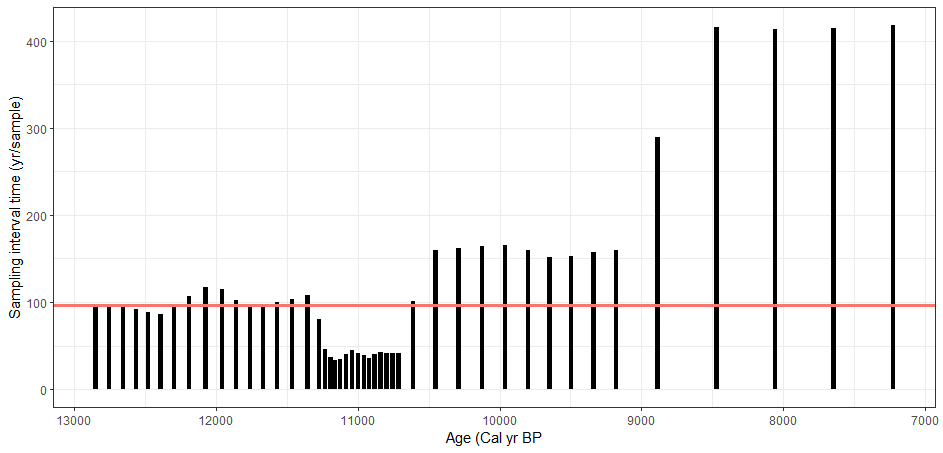
**

**Fig S1. Sampling integration times for the STU (Stump Pond) core.** Plot of the sample integration times (yr/sample) from 13,000-7200 years BP. The red horizontal line indicates the median sampling integration time of 99 years for the entire core.

**
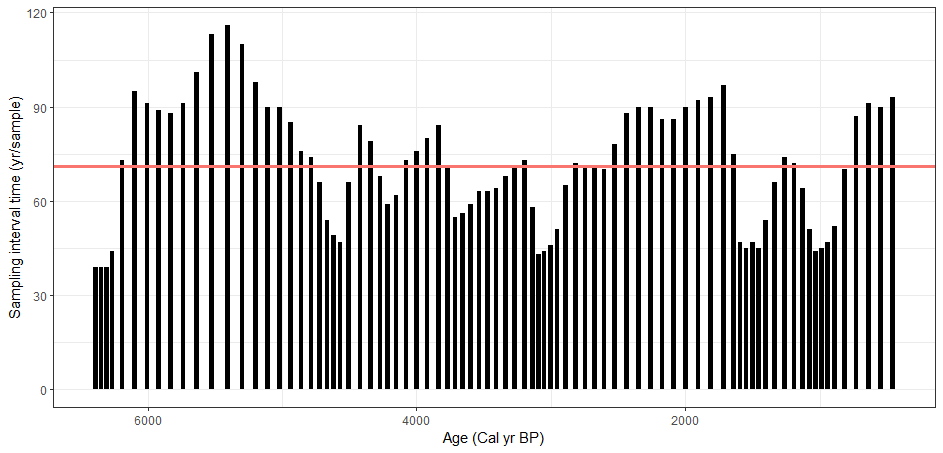
**

**Fig S2. Sampling integration times for the APB1 (wetland) core.** Plot of the sample integration times (yr/sample) from 6400 years BP to present. The red horizontal line indicates the median sampling integration time of 70 years for the entire core.

| 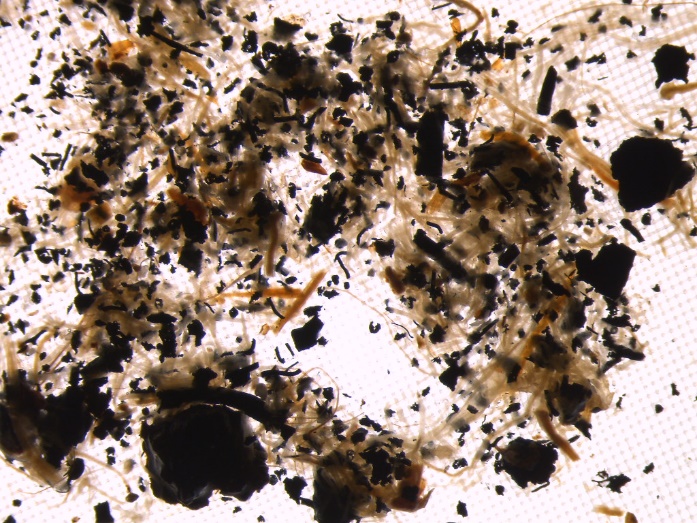 | 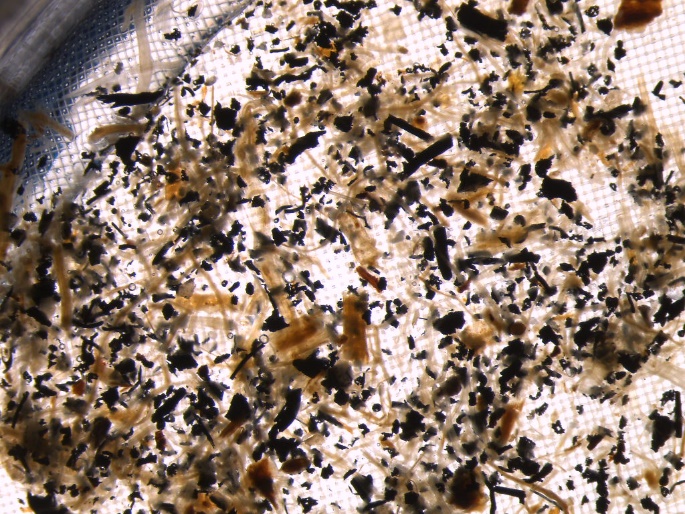 |
| --- | --- |
| 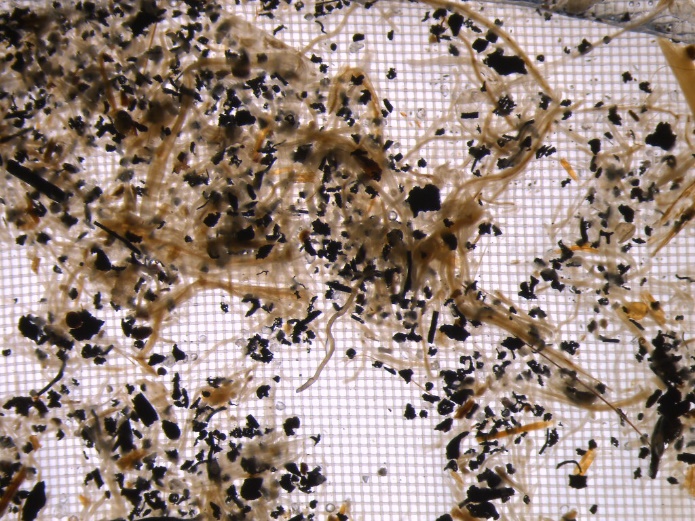 | 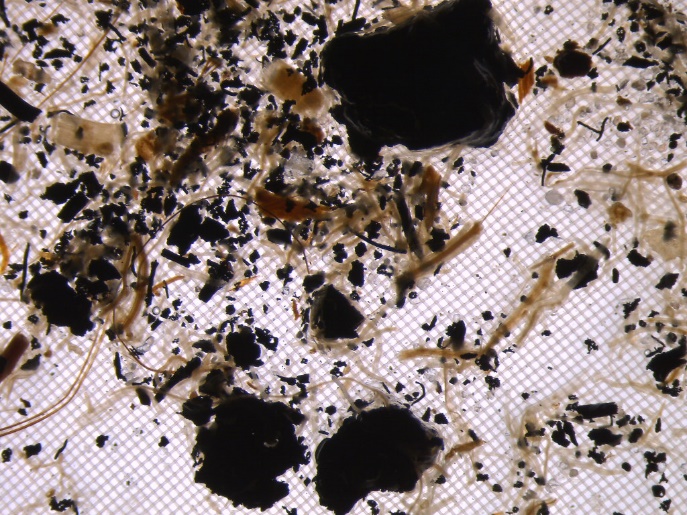 |

**Fig S3. Examples of charcoal content from omitted sample in the APB1 (wetland) core.** The above photographs were taken under the microscope from the sample at a depth of 2-3 cm. Note the large amounts of charcoal present.
